# Risky Alcohol Consumption and Heavy Episodic Drinking among Parents in Germany: Results of a Nationwide Cross-Sectional Study

**DOI:** 10.1155/2019/3673479

**Published:** 2019-12-05

**Authors:** Gianni Varnaccia, Kristin Manz, Johannes Zeiher, Petra Rattay, Cornelia Lange

**Affiliations:** Robert Koch Institute, Department of Epidemiology and Health Monitoring, General-Pape-Str. 62-66, 12101 Berlin, Germany

## Abstract

**Introduction:**

Risky alcohol consumption (RAC) and heavy episodic drinking (HED) by parents can have negative effects on their children. At present, little is known about these forms of alcohol consumption among parents in Germany. The aim of this analysis is to estimate the percentage of parents living in Germany who practise RAC and HED and to study associations between these consumption patterns and sociodemographic factors.

**Material and Methods:**

The data basis comprises the data of the nationwide studies “Gesundheit in Deutschland aktuell” (GEDA) of 2009, 2010, and 2012. The data were collected by means of computer-assisted telephone interviews (CATI). Our analysis included all participants living in a household with at least one child of their own under 18 years of age (*n* = 16,224). Information on RAC and HED was collected using the AUDIT-C screening instrument. Logistic regression models were used to study the relationship between parental alcohol consumption and sociodemographic variables.

**Results:**

18.4% of the mothers and 29.6% of the fathers exhibited RAC; 8.4% of the mothers and 21.0% of the fathers practised HED. After mutual adjustment, RAC showed a significant association with the level of education, income (only mothers), employment status (only mothers), migration background, relationship status (only mothers), and the age of the youngest child. HED showed a significant association with income (only mothers), the age of the youngest child (only mothers), and the level of education (only fathers).

**Conclusions:**

The presented analysis emphasizes the relevance of preventive measures to reduce parental alcohol consumption. In addition to universal interventions, risk group-specific measures (e.g., for parents with high income) are needed to reduce parental alcohol consumption and thus support a healthy development of children.

## 1. Introduction

In addition to the harmful consequences for the drinking individual, excessive alcohol consumption can also have a negative impact on the respective person's environment, especially on close relatives and friends [1–3]. The negative consequences of maternal alcohol consumption during pregnancy and parental alcohol consumption during childhood and adolescence on child development have been studied several times in the past, with particular focus on the children of alcohol-abusing parents [4–7]. Adolescents belong to a group that often reacts particularly sensitively to disturbances in their relationships with reference persons; this can lead to negative long-term consequences for the children and young people. Studies show that children of alcohol-abusing parents themselves develop an addiction to alcohol or some other substance more frequently, that they suffer more frequently from psychological disorders, and that their academic performance is more frequently lower than among children whose parents do not abuse alcohol [5–7].

Less is known about how children and adolescents are affected by parental alcohol consumption that does not necessarily amount to alcohol abuse or dependency [8]. Studies have come to the conclusion that certain parental alcohol consumption patterns, e.g., risky alcohol consumption (RAC) and heavy episodic drinking (HED), can be associated with harmful outcomes for the children, such as high alcohol consumption in later life and physical abuse [8–13]. Furthermore, it was observed that adolescents who have seen their parents under the influence of alcohol are more likely to drink alcohol than young people who have never seen their parents drunk [14]. A study from Germany showed that frequent alcohol consumption by the parents of 12-year-old children is positively associated with heavy episodic drinking by the children in young adulthood [15].

Explanations for these associations include parental role modelling and other parenting factors that can be affected by alcohol consumption [16–18]. For example, alcohol consumption often involves emotional loss of control and inappropriate reactions in certain situations, which can have a negative impact on any children who are present. Moreover, parents recuperating from the consequences of intoxication are usually not in a position to look after their children properly.

To date, only few studies have focused on the prevalence and correlates of parental alcohol consumption patterns based on definitions of drinking that do not necessarily amount to abuse or addiction [19–21]. Thus, the aim of this study is to estimate the proportion of mothers and fathers in Germany who live together with children and adolescents and exhibit RAC or HED. In addition, the study aims to describe sociodemographic characteristics of risk groups in order to identify starting points for target group-specific preventive measures.

## 2. Materials and Methods

### 2.1. Study Design and Participants

Data were obtained from the cross-sectional study “Gesundheit in Deutschland aktuell” (GEDA), a national telephone health interview survey among adults living in Germany [22]. GEDA is part of the nationwide health monitoring system administered by the Robert Koch Institute (RKI). The RKI is a federal institution within the portfolio of the German Federal Ministry of Health responsible for disease control and prevention. The aim of the regular GEDA surveys is to provide current data on population health, health determinants, and the use of health services for national and European health reporting systems, health policies, and public health research.

**Figure 1:**
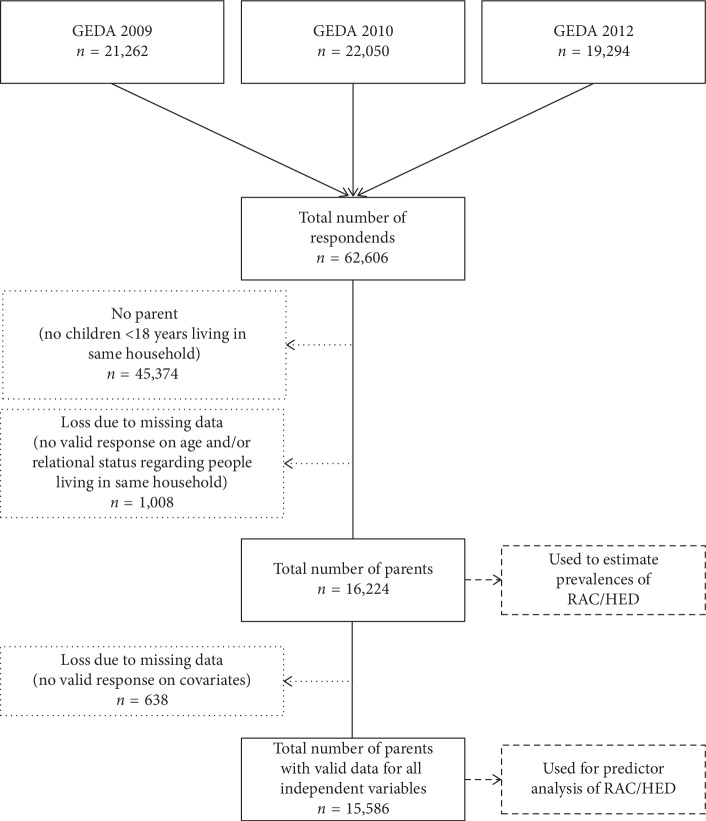


In our analysis, we used pooled data from the GEDA studies conducted in 2009, 2010, and 2012 (see Figure 1). Data were collected by standardized computer-assisted telephone interviewing (CATI). Participants were selected using a two-stage sampling procedure: telephone numbers of households were generated using a random digit dialling procedure, and individuals within the household were selected by the “last birthday method” (GEDA 2009, 2010) or by the Kish Selection Grid (GEDA 2012) [22]. In total, 62,606 people over the age of 18 were questioned between July 2008 and June 2009 (GEDA 2009), between September 2009 and July 2010 (GEDA 2010) and between February 2012 and March 2013 (GEDA 2012). There was no upper age restriction. The response rate (i.e., the number of interviews conducted as a percentage of the estimated number of households in the population), calculated according to the standards of the American Association for Public Opinion Research [23], was 29.1% for GEDA 2009, 28.9% for GEDA 2010, and 22.1% for GEDA 2012. The response rates are comparable to response rates in similar surveys, and a weighted analysis was used to allow inference to the general population [22, 24]. The study was approved by the Federal Commissioner for Data Protection and Freedom of Information, and verbal informed consent was obtained from all the participants in advance. Further information on the design, contents, survey metrics, and results of the GEDA study can be found elsewhere [22]. The analyses were limited to respondents who stated that they were living with at least one child of their own under 18 in the same household (*n* = 16,224, see Figure 1).

No distinction was made between biological, adopted, and step children. In GEDA 2009 and GEDA 2010, parents were identified using three items in the questionnaire: the number of people living in the same household, their ages, and their relationship to the respondent (e.g., daughter or son). In GEDA 2012, the last item was replaced with a question that assessed whether the person under 18 was the respondent's biological, adopted, or step child. Participants whose parental status could not be determined due to a lack of data were excluded from the analysis (*n* = 1,008). The key features of the sample are shown in Table 1. The sample includes data of 9.831 mothers and 6.393 fathers. The mean age of the parents was 40.3 years, and the age range was 18 to 83 years. A comparison between the sample used and the participants with no valid information on parental status can be found in the Supplementary [Supplementary-material supplementary-material-1].

## 3. Variables

### 3.1. Parental Consumption of Alcohol

AUDIT-C (Alcohol Use Disorder Identification Test-Consumption) was used to identify parents with RAC [25–28]. AUDIT-C asks questions about the frequency of alcohol consumption (never, once a month or less frequently, 2 to 4 times a month, 2 to 3 times per week, and 4 times or more per week), the number of alcoholic drinks consumed, respectively (1-2, 3-4, 5-6, 7–9, 10, or more), and the frequency of HED, defined as drinking six or more alcoholic drinks on a single occasion (never, less frequently than once a month, once a month, once a week, every day, or almost every day). Points (from 0 to 4) are awarded in ascending order for the answer categories relating to all questions. According to gender-specific thresholds, a dichotomous variable was generated: women are regarded as risk consumers if their score from the three core questions totals ≥4; the threshold among men is ≥5. In a validation study with a sample of people living in Germany, 74% of the risk consumers (sensitivity) and 83% of the nonrisk consumers (specificity) were correctly identified using AUDIT-C and a threshold of ≥5 [29]. Other studies confirm this result [26, 30].

HED was measured on the basis of the third question in AUDIT-C. People are said to practise HED if they drink six or more alcoholic drinks on a single occasion at least once a month. According to this threshold, a dichotomous variable was generated.

### 3.2. Sociodemographic Variables

In addition to the age of the participants, the analysis took into account the following independent variables: education, income, employment status, experienced unemployment, migration background, partnership, the number of children in the household, and the age of the youngest child in the household.

The participants' level of education was categorized in three groups: “low education level,” “middle education level,” and “high education level,” based on the information provided on the highest school-leaving and vocational qualifications reached according to the CASMIN (Comparative Analysis of Social Mobility in Industrial Nations) classification [31].

The respondents' income situation was measured on the basis of the net equivalent income. A person's (self-reported) income is determined by dividing the total income of all the people living in the household by a weighted sum of the household members. The weighting is carried out according to the new OECD equivalence scale, which assigns the factor 1.0 to the principal earner and the factor 0.5 (≥14 years) or 0.3 (<14 years) to all the other people in the household [32]. Missing income figures were imputed based on age, education, occupational status, and mean regional household net income by a multiple regression process [33]. Three income groups were formed based on the median net equivalent income (€1,375) under 60%, 60 to 150%, and over 150% of the median net equivalent income.

To measure a person's current employment status (self-reported) [34], we differentiate between “employed full-time,” “employed part-time,” and “nonemployed.” The category “nonemployed” includes not only unemployed individuals, but also homemakers, students, and pensioners.

Information on experienced unemployment over the past five years was collected on the basis of several questions. First, the participants were asked whether they had been unemployed in the last five years. If the answer was “yes,” they were asked to state the total duration of unemployment. For the analysis, the participants were divided into two groups: parents who had been unemployed for at least 12 months in the last five years and parents who had been unemployed for less than 12 months or not at all in the last five years.

Migration background was determined from the information provided by respondents on their country of birth or their parents' country of birth. A person was said to have a migration background if she/he herself/himself or at least one of the parents was born abroad.

Other questions related to whether the respondent lived together with a partner, to the number of children in the household, and the age of the youngest child in the household. The number of children in the household was covered by three categories (1 child/2 children/3 children or more). The variable relating to the “age of the youngest child in the household” was also divided into three categories (0–6/7–13/14–17).

### 3.3. Statistical Analysis

To begin with, the percentages of mothers and fathers practising RAC and/or HED, differentiated according to sociodemographic variables, were calculated in the descriptive analysis (with 95% confidence intervals). The results were checked for statistically significant differences (*p* < 0.05) using Pearson's *χ*2 tests, which were corrected according to Rao and Scott and converted into an F statistic. Using logistic regressions, the associations between the outcomes (RAC and/or HED) and the independent variables (age, education, income, employment status, experienced unemployment, migration background, relationship status, number of children living in the household, and age of the youngest child) were subsequently examined multivariately. In the first step, age-adjusted odds ratios (ORs) were calculated for all the abovedescribed independent variables in separate models for RAC and HED. In the second step, all the variables were taken into account in the model at the same time. All analyses were conducted separately for mothers and fathers in order to identify possible gender differences in parental drinking behaviour. The evaluations involved the use of weighting factors to take into account the two-stage sampling design, and the sample was adjusted to reflect Germany's resident population in terms of age, gender, education, and regional distribution [22]. All analyses were conducted using Stata 14.1 survey design procedures.

## 4. Results

### 4.1. Maternal Risky Alcohol Consumption

Table 2 shows prevalences of RAC in mothers according to sociodemographic factors. 18.4% of the mothers reported RAC. Bivariate analyses revealed that maternal RAC was associated with age, education, income, employment status, experienced unemployment, migration background, living with a partner, the number of children, and the age of the youngest child. RAC was less common among younger mothers compared with older mothers (increasing from 13.3% in the 18–29 age group to 23.9% in the 50+ age group). Mothers with a low education level had lower rates of RAC (12.4%) than mothers with middle (19.1%) or high education (23.1%) levels. Mothers with a low income showed lower rates of RAC (11.9%) than mothers in middle (18.8%) or high income groups (23.3%). Furthermore, part-time (20.8%) and full-time (20.1%) employed mothers had higher rates of RAC than nonemployed mothers (12.9%). RAC was less prevalent among mothers who had experienced a longer period of unemployment than among mothers who had not (13.7% vs. 19.0%). Lower rates of RAC were observed among mothers with a migration background than among mothers without a migration background (12.2% vs. 20.4%). Mothers living without a partner had higher rates of RAC than mothers living with a partner (21.1% vs. 17.9%). As regards the number of children, mothers with three or more children showed lower rates of RAC (14.2%) than mothers with one child (19.2%) or two children (18.8%), and mothers with children aged 0–6 showed lower rates of RAC (14.4%) than mothers whose youngest child was aged 7–13 (20.9%) or 14–17 (22.6%).

Calculating age-adjusted ORs for RAC revealed that age, education, income, employment status, experienced unemployment, migration background, number of children, and age of the youngest child were significantly associated with RAC among mothers (see Table 3).

In the multivariate analysis (all independent variables included), mothers with a middle or high education level showed higher odds for RAC than mothers with low education (OR: 1.42 and 1.76). Among mothers in the middle or high income group, the odds for RAC were higher than among mothers with a low income (OR: 1.46 and 1.71). Furthermore, part-time or full-time employed mothers had higher odds for RAC than nonemployed mothers (OR: 1.40 and 1.27). Mothers with a migration background showed lower odds (OR 0.63) for RAC than mothers without a migration background. In addition, mothers living without a partner were significantly more likely to report RAC than mothers who lived with a partner (OR 1.26). Compared with mothers with children aged 0–6, odds of RAC were higher in mothers whose youngest child was aged 7–13 (OR 1.39) or 14–17 (OR 1.44).

### 4.2. Maternal Heavy Episodic Drinking

Table 2 shows prevalences of HED in mothers according to sociodemographic factors. In total, 8.4% of the mothers reported consuming six or more alcoholic drinks on a single occasion at least once a month. In bivariate analyses, maternal HED was associated with age, education, income, employment status, experienced unemployment, migration background, the number of children, and the age of the youngest child living in the household. Mothers in the 18–29 (7.4%) and 30–39 (7.2%) age groups had lower rates of HED than mothers in the 40–49 (9.8%) and 50+ (8.9%) age groups. As regards the level of education, the highest rates of HED were observed in mothers with a middle education level (9.2%). HED was less common among mothers in low income groups (5.9%) than among mothers in middle (8.9%) or high income groups (9.3%). Furthermore, nonemployed mothers showed lower HED rates (6.3%) than part-time (9.5%) and full-time employed mothers (8.9%). Mothers who had experienced a longer period of unemployment showed lower rates of HED than mothers who had not (5.9% vs. 8.8%), and mothers with a migration background had lower rates of HED than mothers with no migration background (6.8% vs. 9.1%). As regards the number of children living in the household, the lowest rates of HED were observed in mothers with three or more children (6.7%). Mothers with a child aged 0–6 had a lower prevalence of HED (6.7%) than mothers whose youngest child was aged 7–13 (9.4%) or 14–17 (10.6%).

Table 4 shows age-adjusted odds ratios for maternal HED and the covariates considered. Income, employment status, experienced unemployment, migration background, number of children in the household, and age of the youngest child were significantly associated with HED in mothers.

The multivariate analysis revealed that mothers in middle and high income groups had higher odds of HED than mothers in low income groups (OR: 1.43 and 1.49). Furthermore, mothers whose youngest child was aged 7–13 or 14–17 had higher odds of HED than mothers with children aged 0–6 (OR: 1.36 and 1.45).

### 4.3. Paternal Risky Alcohol Consumption

Table 2 shows prevalences of RAC in fathers according to sociodemographic factors. 29.6% of the fathers reported RAC. Bivariate analyses revealed that paternal RAC was associated with education, income, experienced unemployment, migration background, and the age of the youngest child. Fathers with a low education level had lower prevalences of RAC than fathers with middle education (26.6% vs. 32.2%). Lower rates of RAC were also observed among fathers in low income groups (23.6%) compared with fathers in middle (30.0%) and high income groups (32.4%). Fathers who had experienced a longer period of unemployment had lower RAC rates than fathers who had not (22.7% vs. 30.1%). Fathers with a migration background showed lower rates of RAC than fathers without a migration background (23.5% vs. 31.6%). Furthermore, fathers living with a child aged 0–6 had lower rates of RAC than fathers whose youngest child was aged 14–17 (27.4% vs. 34.3%).

After adjusting for age, the independent variables of education, income, migration background, number of children living in the household, and age of the youngest child were associated with RAC in fathers (see Table 3).

The multivariate analysis revealed higher odds for RAC in fathers with a middle education level (OR 1.22) compared with fathers with low education. Furthermore, fathers with a migration background had lower odds for RAC (OR 0.72) than fathers without a migration background. As regards the age of the youngest child in the household, the higher odds for RAC in fathers whose youngest child was aged 14–17 (OR 1.33, reference group: 0–6) also persisted in the multivariate logistic model.

### 4.4. Paternal Heavy Episodic Drinking

Table 2 shows prevalences of HED in fathers according to sociodemographic factors. 21.0% of the fathers reported consuming six or more alcoholic drinks on a single occasion at least once a month. In bivariate analyses, paternal HED was associated with education, income, and number of children living in the household. Among fathers with a low education level, HED was less common than among fathers with middle education (18.0% vs. 23.7%). Furthermore, fathers in low income groups (16.1%) showed lower rates of HED than fathers in middle (21.5%) and high income groups (22.7%). As regards the number of children living in the household, the lowest rates of HED were observed among fathers living with three or more children (16.9%). After adjusting for age, HED in fathers was associated with education and income (see Table 4). The multivariate analysis revealed that fathers with middle education had higher odds of HED than fathers with low education (OR: 1.38).

## 5. Discussion

In this nationwide study, almost a fifth of mothers of children under the age of 18 and almost a third of fathers reported RAC. Approximately one tenth of the mothers and about a fifth of the fathers practised HED, i.e., drank six or more alcoholic beverages on a single occasion at least once a month. Multivariate analysis showed that a low level of education, a migration background, and living with a young child in the household were negatively associated with parental RAC. Higher incomes, employment, and living without a partner were positively associated with RAC only among mothers. In the case of HED, income and living without a young child in the household were positively associated with the outcome among mothers. Among fathers, a negative association between a low education and HED was observed.

### 5.1. Possible Explanations for Sociodemographic Differences in Parental RAC and HED

The lower alcohol consumption among mothers compared with fathers could be partly due to the fact that higher alcohol consumption tends to be disapproved by society more when practised by women than by men [35]. This probably applies especially to mothers. Furthermore, biological factors also play a role, e.g., the same quantity of alcohol consumed leads to a higher concentration of alcohol in the blood of women [35]. A large number of studies confirm higher alcohol consumption among fathers compared with mothers [19, 21, 36–38].

Fathers and mothers with a higher level of education were more likely to practise RAC than parents with a low education level; fathers with a higher level of education practised HED more frequently than fathers with a low level of education. In the case of mothers, higher income was connected with RAC and HED. Possibly, more frequent RAC or HED among parents in high social strata can be explained by a status-specific habitus that is characterized partly by their working environment and social network. Another possible explanation approach in the case of mothers might be a departure from traditional role images in high social-status groups. Further studies that have investigated the link between parental alcohol consumption and the level of education have arrived at contradictory findings [21, 38].

Nonemployed mothers showed lower rates of RAC than employed mothers. Kuntsche et al. [39] has found that the association between mothers' employment status and maternal alcohol use varied between countries with high versus low gender-income equity; in the Nordic countries with high gender-income equity, partnered mothers who engaged in paid labour drank less alcohol per occasion, whereas alcohol use was higher among partnered mothers working for pay in countries with lower gender-income equity like Germany or Switzerland. The authors argued that the combination of motherhood and employment may be a source of conflict for women in Switzerland and Germany, where a more traditional role model of being a mother is favoured. Therefore, in Germany, paid labour may constitute an additional source of stress or overload (work-family conflict), which might lead to the use of alcohol as a coping strategy. However, it would also be quite conceivable that in Germany high alcohol consumption is not in line with the traditional role model of the mother, while the role models and drinking habits of working mothers tend to be more similar to economically active childless women.

Mothers and fathers with a migration background practised RAC less often than parents without a migration background. Sociocultural aspects are sometimes offered as one possible explanation (among others) for migration-specific differences in parental alcohol consumption [40]. For example, the lower alcohol consumption of parents with a migration background compared with parents without a migration background might be explained in part by the fact that a large proportion of people with a migration background come from Islamic countries [41], where the consumption of alcohol is substantially less common or even banned. Differences in alcohol consumption between parents with different cultural origins have also been observed in the United States of America [21].

Mothers living with their child without a partner reported RAC more frequently than mothers living in a partnership. Higher alcohol consumption by single mothers compared to mothers in a partnership could be explained both by more stress in everyday life and more frequent social activities where alcohol is consumed. The results of the Canadian National Population Health Survey (NPHS) confirm that mothers living alone exhibit HED more frequently than those who live in a partnership [42]. In the Australian National Drug Strategy Household Survey (NDSHS), it was observed that a parent who lives alone is more likely to practise HED than one living in a partner relationship [19]. However, the results of the National Health Survey in Australia were only able to confirm this for mothers living alone. The percentage of fathers who practise HED was lower among single fathers than among fathers living in a partnership [19].

Fathers and mothers living with younger children in the household showed signs of RAC less frequently than parents living with exclusively older children. Furthermore, mothers with younger children reported HED less often than mothers with exclusively older children. The smaller percentage of risk consumers among parents of younger children compared to parents living exclusively with older children in a household could be explained by the fact that parents, especially mothers, reduce their alcohol consumption during pregnancy and in the first years of the child's life [43], but return to more common alcohol consumption levels in the population as the child grows older. The results of further studies on the relationship between the child's age and the parents' alcohol consumption are contradictory. While an Australian study has confirmed the results obtained for RAC and HED in mothers, a Swiss study observed no link in mothers or fathers [19, 38].

### 5.2. Comparing the Results with Observations in the General Population

Most of the reported differences in the parents' alcohol consumption can also be observed in the general population. For example, women drink less alcohol than men [44–46], people with a low level of education less than people with a higher education level [44, 46, 47], and people with low income less than people with high income [46]. Among women, long weekly working hours are positively associated with alcohol consumption [46]. With regard to migration background, people from Turkey, which represents the most common foreign country of origin in Germany, have significantly higher abstinence rates than people with no migration background [46, 48]. Thus, our study suggests that correlates of alcohol consumption do not differ substantially between parents and nonparents in the general population, with one notable exception. Prevalence of RAC and HED were significantly lower among parents than nonparents, especially in younger age groups and for women, so that for women, age was directly related to RAC and HED among parents but inversely related to these behaviours in women who were not parents (see Supplementary [Supplementary-material supplementary-material-1]). Furthermore, it should be mentioned that alcohol consumption levels in Germany are well above the international/EU average [49].

## 6. Limitations

The aim of this paper was to estimate the prevalence of RAC and HED among parents in Germany and to identify associations between these consumption patterns and sociodemographic factors. Based on this, it would be interesting to analyse associations with adverse childhood outcomes and to identify theory-guided possible mechanisms such as parental modelling and other parenting factors. However, we were not able to perform such analyses, as GEDA does not include information on the children or possible mechanisms. Further limitations of this analysis are mainly due to the study design of GEDA. Because GEDA is a cross-sectional study, no causal conclusions can be drawn. Furthermore, GEDA is a landline-based telephone survey carried out in German language. As a result, certain population groups are underrepresented in the sample. These include people who can only be contacted via mobile phones or have insufficient German language skills. Telephone surveys in general are prone to biases like social desirability or interviewer effects, which should be taken into account when interpreting the results presented [50]. Furthermore, low response rates are a common problem in telephone surveys [24]. In order to allow representative analysis, a complex weighting procedure was applied in our study [22]. As regards the assessment of alcohol consumption, it was observed that the reported alcohol consumption tends to be lower in telephone surveys than in surveys that use self-administered questionnaires [51]. Furthermore, validation studies using the AUDIT-C definitions show some misclassification for both sensitivity and specificity, which may also contribute to bias in the results. Despite these limitations, GEDA provides a nationwide random sample that delivers information on parents of children under the age of 18 (including those living in one-parent families), enabling a comprehensive description of self-reported parental alcohol consumption in Germany.

The comparability of the presented results with other studies is limited due to methodological and cultural issues. Considering the methodological issues, comparisons are limited through different study designs, outcomes, and instruments. To assess RAC, we used the AUDIT-C instrument. Other studies used different instruments like the CAGE-C [36]. HED was defined as drinking six or more alcoholic beverages on a single occasion at least once a month. Other studies defined HED differently, e.g., as having ≥7 drinks for men and ≥5 drinks for women on one occasion [19]. Using the AUDIT-C to identify parents with RAC and a cut-off score of six alcoholic drinks for HED might have led to higher prevalence rates compared with studies that used less sensitive instruments for RAC and higher cut-off scores for HED. While our study is based on a nationwide representative sample, other studies collected data differently, e.g., from parents of children in paediatric clinics [36, 37]. Considering the cultural reasons for the limited comparability of the presented results, different alcohol consumption patterns and cultural norms have to be mentioned. Firstly, alcohol consumption patterns largely differ between countries [52]. Thus, parental alcohol consumption might be influenced by traditional drinking behaviours. Secondly, cultural norms might affect the social disapproval of alcohol consumption in general among men and women but also particularly among fathers and mothers. Furthermore, socially established role models might play a role when comparing parental alcohol consumption patterns between countries [39].

## 7. Conclusion

RAC and HED are prevalent among fathers and mothers in Germany. It has been observed in several studies that parental alcohol consumption which need not amount to alcohol abuse can have negative consequences for children [8–13]. Since RAC and HED also exist among well-integrated parents and, unlike alcohol dependence, are not primarily associated with a lack of social participation opportunities, what is needed most are universal preventive measures that address the entire population (e.g., measures aimed at fostering a culture in which social events are not necessarily linked to alcohol consumption). In addition, selective measures should be promoted which target parents in risk groups and especially relevant life phases. Considering the elevated rates of RAC and HED among parents in high social strata, target group-specific measures such as brief counselling or medical advice in primary health care settings could be an effective preventive approach [53]. To reach employed parents and, depending on the social status of the employees, parents in high social strata, workplace interventions might also be an appropriate approach to reduce parental alcohol consumption [54]. Since many parents-to-be are prepared to lead a healthy lifestyle and change their behaviour, e.g., during pregnancy, preventive measures should support them in this life phase [55]. Moreover, health-promotion activities should help parents to also maintain a healthy lifestyle when their children grow older. When it comes to raising awareness for a less risky attitude to alcohol, “early intervention” initiatives for parents, as well as doctors, educators, teachers, and trainers in sports clubs, could play an important role. Furthermore, initiatives like national health targets might provide a framework for preventive activities aiming to reduce alcohol consumption among parents. For instance, the German health target “reduce alcohol consumption” aims to increase the number of women who do not drink alcohol during pregnancy and lactation, while men are also encouraged to reduce alcohol consumption during the transition to parenthood [55]. Further studies should analyse the impact of parental RAC and HED on children's health behaviour, identify theory-guided possible mechanisms, and investigate which strategies are the most effective at reaching parents with risky alcohol consumption.

## Figures and Tables

**Table 1:** Sample characteristics, mothers, and fathers (aged 18+), GEDA 2009/2010/2012, *n* = 16,224.

	Mothers			Fathers		
	*n*	%	%^a^	*n*	%	%^a^
Age group						
18–29	852	8.7	11.2	272	4.3	5.3
30–39	3,696	37.6	40.6	1,854	29.0	32.0
40–49	4,561	46.4	41.4	3,256	50.9	47.5
50+	722	7.3	6.8	1,011	15.8	15.2
Missing	0	—	—	0	—	—

Education^b^						
Low	1,373	14.0	22.1	1,248	19.5	30.9
Middle	5,839	59.4	59.7	2,762	43.2	45.4
High	2,611	26.6	18.2	2,380	37.2	23.7
Missing	8	0.1	—	3	0.0	—

Income^c^						
Low	1,292	13.1	16.2	532	8.3	12.7
Middle	6,843	69.6	69.5	4,333	67.8	70.1
High	1,696	17.3	14.2	1,528	23.9	17.2
Missing	0	—	—	0	—	—

Employment status						
Nonemployed	2,443	24.8	29.2	344	5.4	7.3
Part time	5,309	54.0	52.1	385	6.0	5.9
Full time	2,044	20.8	18.7	5,652	88.4	86.8
Missing	35	0.4	—	12	0.2	—

Experienced unemployment^d^						
No	8,737	88.9	86.9	6,097	95.4	93.5
Yes	1,083	11.0	13.1	282	4.4	6.5
Missing	11	0.2	—	14	0.2	—

Migration background						
No	7,937	80.7	79.2	5,248	82.1	80.1
Yes	1,588	16.2	20.8	915	14.3	19.9
Missing	306	3.1	—	230	3.6	—

Living with partner						
No	2,076	21.1	14.3	253	4.0	2.5
Yes	7,739	78.7	85.7	6,135	96.0	97.5
Missing	16	0.2	—	5	0.1	—

Children in household						
1	4,590	46.7	47.1	2,881	45.1	46.8
2	4,060	41.3	39.7	2,746	43.0	41.2
3+	1,181	12.0	13.2	766	12.0	12.0
Missing	0	—	—	0	—	—

Age of the youngest child						
0–6	4,109	41.8	43.9	2,722	42.6	42.7
7–13	3,868	39.3	37.1	2,445	38.2	37.0
14–17	1,854	18.9	18.9	1,226	19.2	20.3
Missing	0	—	—	0	—	—

Risky alcohol consumption^e^						
No	7,788	79.2	81.6	4,450	69.6	70.4
Yes	1,977	20.1	18.4	1,913	29.9	29.6
Missing	66	0.7	—	30	0.5	—

Heavy episodic drinking^f^						
No	8,943	91.0	91.6	5,012	78.4	79.0
Yes	858	8.7	8.4	1,369	21.4	21.0
Missing	30	0.3	—	12	0.2	—

Study						
GEDA09	3,763	38.3	38.8	2,115	33.1	33.6
GEDA10	3,836	39.0	38.8	2,271	35.5	35.1
GEDA12	2,232	22.7	22.3	2,007	31.4	31.3

^a^weighted by sex, age, federal state, and education for the German population on 31 December 2011 (without missing values); ^b^according to the CASMIN classification; ^c^net equivalent income compared to the median net equivalent income (low: under 60%, middle: 60–150%, and high: >150%); ^d^12 months or more in the last 5 years; ^e^AUDIT-C score (mothers: ≥4; fathers: ≥5); ^f^≥6 alcoholic drinks on a single occasion at least once a month.

**Table 2:** Prevalence of risky alcohol consumption and heavy episodic drinking among mothers and fathers according to selected covariates, *n* = 16,224.

	Risky alcohol consumption^a^				Heavy episodic drinking^b^			
	Mothers		Fathers		Mothers		Fathers	
	%	95% CI	%	95% CI	%	95% CI	%	95% CI
Total	18.4	(17.5–19.3)	29.6	(28.2–31.0)	8.4	(7.8–9.1)	21.0	(19.8–22.2)

Age group								
18–29	13.3	(10.9–16.2)	27.4	(21.8–33.9)	7.4	(5.6–9.8)	22.4	(17.3–28.6)
30–39	15.5	(14.2–16.9)	28.4	(26.0–30.9)	7.2	(6.3–8.3)	21.7	(19.5–24.0)
40–49	21.6	(20.3–23.1)	29.6	(27.7–31.5)	9.8	(8.8–10.9)	21.1	(19.4–22.8)
50+	23.9	(20.2–28.0)	32.9	(29.3–36.8)	8.9	(6.7–11.7)	18.8	(16.0–22.1)
*p* value	<0.001		0.190		0.005		0.483	

Education^c^								
Low	12.4	(10.4–14.7)	26.6	(23.8–29.5)	6.9	(5.5–8.6)	18.0	(15.7–20.6)
Middle	19.1	(18.0–20.3)	32.2	(30.2–34.3)	9.2	(8.4–10.1)	23.7	(21.9–25.6)
High	23.1	(21.4–24.9)	28.4	(26.5–30.4)	7.8	(6.7–9.0)	19.7	(18.1–21.5)
*p* value	<0.001		0.002		0.019		<0.001	

Income^d^								
Low	11.9	(9.9–14.2)	23.6	(19.6–28.2)	5.9	(4.5–7.6)	16.1	(12.8–20.2)
Middle	18.8	(17.8–19.9)	30.0	(28.3–31.6)	8.9	(8.1–9.7)	21.5	(20.1–23.0)
High	23.3	(21.1–25.7)	32.4	(29.7–35.3)	9.3	(7.8–10.9)	22.7	(20.3–25.2)
*p* value	<0.001		0.004		0.003		0.014	

Employment status								
Nonemployed	12.9	(11.4–14.6)	27.2	(21.4–33.9)	6.3	(5.2–7.5)	17.8	(13.0–23.9)
Part time	20.8	(19.6–22.1)	29.0	(23.8–34.7)	9.5	(8.6–10.4)	16.0	(12.0–20.8)
Full time	20.1	(18.1–22.2)	29.9	(28.4–31.3)	8.9	(7.5–10.4)	21.5	(20.3–22.8)
*p* value	<0.001		0.654		<0.001		0.084	

Experienced unemployment^e^								
No	19.0	(18.0–19.9)	30.1	(28.7–31.5)	8.8	(8.1–9.5)	21.4	(20.2–22.6)
Yes	13.7	(11.4–16.4)	22.7	(17.5–28.9)	5.9	(4.4–7.7)	16.3	(11.7–22.1)
*p* value	<0.001		0.024		0.005		0.087	

Migration background								
No	20.4	(19.4–21.5)	31.6	(30.1–33.2)	9.1	(8.4–9.9)	21.7	(20.4–23.0)
Yes	12.2	(10.4–14.3)	23.5	(20.2–27.1)	6.8	(5.5–8.5)	19.4	(16.5–22.7)
*p* value	<0.001		<0.001		0.013		0.195	

Living with partner								
No	21.1	(18.9–23.5)	31.4	(24.8–38.9)	9.8	(8.3–11.5)	24.2	(18.2–31.4)
Yes	17.9	(16.9–18.9)	29.5	(28.1–30.9)	8.2	(7.5–9.0)	20.9	(19.7–22.1)
*p* value	0.009		0.597		0.066		0.308	

Children in household								
1	19.2	(17.9–20.6)	30.7	(28.7–32.8)	9.3	(8.4–10.3)	21.1	(19.4–22.9)
2	18.8	(17.4–20.2)	29.7	(27.6–31.8)	8.0	(7.1–9.0)	22.1	(20.3–24.0)
3+	14.2	(12.0–16.7)	25.0	(21.3–29.2)	6.7	(5.2–8.8)	16.9	(13.8–20.6)
*p* value	0.002		0.051		0.033		0.045	

Age of the youngest child								
0–6	14.4	(13.2–15.6)	27.4	(25.4–29.5)	6.7	(5.9–7.6)	20.9	(19.1–22.7)
7–13	20.9	(19.4–22.5)	29.5	(27.4–31.8)	9.4	(8.3–10.6)	20.8	(18.9–22.9)
14–17	22.6	(20.4–25.0)	34.3	(31.1–37.7)	10.6	(9.1–12.4)	21.6	(18.9–24.5)
*p* value	<0.001		0.001		<0.001		0.896	

^a^AUDIT-C score (mothers: ≥4; fathers: ≥5); ^b^≥6 alcoholic drinks on a single occasion at least once a month; ^c^according to the CASMIN classification; ^d^net equivalent income compared with the median net equivalent income (low: under 60%, middle: 60–150%, high: >150%); ^e^12 months or more in the last 5 years.

**Table 3:** Associations between risky alcohol consumption and selected covariates among mothers and fathers (logistic regression; odds ratios; and figures in bold print, *p* value < 0.05), *n* = 15,497.

	Mothers^a^				Fathers^b^			
	Age-adjusted		All-adjusted		Age-adjusted		All-adjusted	
	OR	95% CI	OR	95% CI	OR	95% CI	OR	95% CI
Age group								
18–29	1		1		1		1	
30–39	1.18	(0.92–1.52)	0.89	(0.68–1.15)	1.00	(0.72–1.40)	0.98	(0.70–1.38)
40–49	**1.77**	**(1.38–2.27)**	1.03	(0.78–1.36)	1.08	(0.78–1.49)	0.95	(0.67–1.34)
50+	**1.92**	**(1.41–2.63)**	1.09	(0.76–1.56)	1.28	(0.90–1.83)	1.06	(0.72–1.57)

Education^c^								
Low	1		1		1		1	
Middle	**1.66**	**(1.34–2.06)**	**1.42**	**(1.14–1.76)**	**1.33**	**(1.11–1.59)**	**1.22**	**(1.02–1.46)**
High	**2.05**	**(1.64–2.56)**	**1.76**	**(1.39–2.22)**	1.07	(0.90–1.28)	0.95	(0.78–1.15)

Income^d^								
Low	1		1		1		1	
Middle	**1.74**	**(1.40–2.15)**	**1.46**	**(1.16–1.84)**	1.30	(1.00–1.68)	1.17	(0.89–1.55)
High	**2.18**	**(1.71-2.78)**	**1.71**	**(1.30-2.25)**	**1.43**	**(1.08–1.90)**	1.35	(0.99–1.85)

Employment status								
Nonemployed	1		1		1		1	
Part time	**1.69**	**(1.43–1.98)**	**1.40**	**(1.18–1.66)**	1.07	(0.71–1.63)	1.01	(0.66–1.54)
Full time	**1.64**	**(1.35–1.99)**	**1.27**	**(1.03–1.56)**	1.09	(0.79–1.52)	0.93	(0.66–1.30)

Experienced unemployment^e^								
No	1		1		1		1	
Yes	**0.71**	**(0.57–0.88)**	0.92	(0.73–1.16)	0.72	(0.51–1.02)	0.82	(0.57–1.18)

Migration background								
No	1		1		1		1	
Yes	**0.57**	**(0.47–0.68)**	**0.63**	**(0.52–0.76)**	**0.67**	**(0.55–0.83)**	**0.72**	**(0.58–0.89)**

Living with partner								
Yes	1		1		1		1	
No	1.16	(0.99–1.35)	**1.26**	**(1.07–1.49)**	1.11	(0.79–1.55)	1.05	(0.75–1.48)

Children in household								
1	1		1		1		1	
2	1.02	(0.90–1.16)	1.09	(0.95–1.25)	0.95	(0.82–1.09)	1.02	(0.88–1.19)
3+	**0.74**	**(0.60–0.92)**	0.94	(0.75–1.18)	**0.78**	**(0.61–0.99)**	0.93	(0.73–1.19)

Age of the youngest child								
0–6	1		1		1		1	
7–13	**1.39**	**(1.19–1.61)**	**1.39**	**(1.19–1.63)**	1.06	(0.90–1.25)	1.05	(0.89–1.24)
14–17	**1.38**	**(1.13–1.68)**	**1.44**	**(1.16–1.78)**	**1.35**	**(1.10–1.67)**	**1.33**	**(1.06–1.66)**

^a^AUDIT-C score ≥4; ^b^AUDIT-C score ≥5; ^c^according to the CASMIN classification; ^d^net equivalent income compared with the median net equivalent income (low: under 60%, middle: 60–150%, high: >150%); ^e^12 months or more in the last 5 years.

**Table 4:** Associations between heavy episodic drinking and selected covariates among mothers and fathers (logistic regression; odds ratios; and figures in bold print, *p* value <0.05), *n* = 15,547.

	Mothers^a^				Fathers^a^			
	Age-adjusted		All-adjusted		Age-adjusted		All-adjusted	
	OR	95% CI	OR	95% CI	OR	95% CI	OR	95% CI
Age group								
18–29	1		1		1		1	
30–39	0.97	(0.69–1.35)	0.84	(0.59–1.19)	0.89	(0.62–1.28)	0.89	(0.62–1.28)
40–49	1.32	(0.96–1.83)	0.91	(0.63–1.31)	0.91	(0.64–1.28)	0.87	(0.60–1.27)
50+	1.16	(0.75–1.81)	0.74	(0.45–1.24)	0.82	(0.55–1.21)	0.79	(0.51–1.23)

Education^b^								
Low	1		1		1		1	
Middle	1.28	(0.98–1.68)	1.12	(0.85–1.48)	**1.45**	**(1.19–1.77)**	**1.38**	**(1.12–1.69)**
High	1.05	(0.78–1.41)	0.94	(0.69–1.28)	1.16	(0.94–1.42)	1.07	(0.86–1.33)

Income^c^								
Low	1		1		1		1	
Middle	**1.56**	**(1.16–2.10)**	**1.43**	**(1.05–1.97)**	**1.37**	**(1.02–1.85)**	1.25	(0.90–1.72)
High	**1.57**	**(1.12–2.19)**	**1.49**	**(1.03–2.15)**	**1.48**	**(1.07–2.03)**	1.39	(0.97–1.99)

Employment status								
Nonemployed	1		1		1		1	
Part time	**1.46**	**(1.17–1.82)**	1.25	(0.98–1.59)	0.80	(0.49–1.32)	0.74	(0.44–1.23)
Full time	**1.37**	**(1.05–1.80)**	1.10	(0.81–1.48)	1.14	(0.78–1.66)	0.99	(0.66–1.48)

Experienced unemployment^d^								
No	1		1		1		1	
Yes	**0.69**	**(0.50–0.94)**	0.77	(0.56–1.08)	0.76	(0.51–1.13)	0.91	(0.60–1.40)

Migration background								
No	1		1		1		1	
Yes	**0.75**	**(0.59–0.96)**	0.81	(0.63–1.05)	0.82	(0.66–1.02)	0.93	(0.75–1.16)

Living with partner								
Yes	1		1		1		1	
No	1.18	(0.96–1.45)	1.21	(0.97–1.50)	1.23	(0.85–1.78)	1.25	(0.85–1.83)

Children in household								
1	1		1		1		1	
2	0.86	(0.71–1.03)	0.91	(0.75–1.10)	1.03	(0.88–1.20)	1.08	(0.92–1.26)
3+	**0.72**	**(0.53–0.98)**	0.88	(0.64–1.22)	0.79	(0.60–1.02)	0.88	(0.67–1.15)

Age of the youngest child								
0–6	1		1		1		1	
7–13	**1.44**	**(1.16–1.79)**	**1.36**	**(1.07–1.71)**	1.02	(0.85–1.21)	0.99	(0.83–1.19)
14–17	**1.61**	**(1.21–2.13)**	**1.45**	**(1.06–1.97)**	1.15	(0.91–1.45)	1.13	(0.88–1.44)

^a^≥6 alcoholic drinks on a single occasion at least once a month; ^b^according to the CASMIN classification; ^c^net equivalent income compared with the median net equivalent income (low: under 60%, middle: 60–150%, high: >150%); ^d^12 months or more in the last 5 years.

## Data Availability

The datasets analysed in this document are available upon request in the repository of the Robert Koch Institute (https://www.rki.de/DE/Content/Gesundheitsmonitoring/Forschungsdatenzentrum/forschungsdatenzentrum_inhalt.html).

## Supplementary Materials

Supplementary Materials Supplementary material 1: characteristics of included and excluded cases according to the selected sociodemographic variables, GEDA 2009/2010/2012, *n* = 17,232. Supplementary material 2: prevalence of risky alcohol consumption and heavy episodic drinking among nonparents according to selected covariates, *n* = 45,203.
