# Supplementary material for: Risky Alcohol Consumption and Heavy Episodic Drinking among Parents in Germany: Results of a Nationwide Cross-Sectional Study
Source: J Environ Public Health. 2019 Dec 5;2019:3673479. doi: 10.1155/2019/3673479 (PMC6915141; doi:10.1155/2019/3673479)
Supplement: Supplementary Materials — Supplementary material 1: characteristics of included and excluded cases according to the selected sociodemographic variables, GEDA 2009/2010/2012, n = 17,232. Supplementary material 2: prevalence of risky alcohol consumption and heavy episodic drinking among nonparents according to selected covariates, n = 45,203. [file 3673479.f1.pdf]

Supplementary material 1: Characteristics of included and excluded cases according to selected sociodemographic variables, GEDA 2009/2010/2012, n=17,232

	Included cases (n=16,224)		Excluded cases (n=1,008)		p-value
	%	95% CI	%	95% CI	
**Sex**					
Female	52.9	(52.0-53.9)	39.0	(35.5-42.7)	
Male	47.1	(46.1-48.0)	61.0	(57.3-64.5)	<0.001
**Age group**					
18-29	8.4	(7.9-9.0)	31.9	(28.5-35.4)	
30-39	36.6	(35.7-37.5)	18.4	(15.6-21.5)	
40-49	44.3	(43.3-45.2)	25.1	(22.0-28.5)	
50+	10.8	(10.2-11.4)	24.6	(21.4-28.2)	<0.001
**Education**[a]					
Low	26.3	(25.3-27.2)	32.7	(28.9-36.8)	
Middle	52.9	(52.0-53.9)	49.3	(45.4-53.2)	
High	20.8	(20.2-21.4)	18.0	(15.8-20.4)	0.108
**Income**[b]					
Low	14.6	(13.8-15.4)	23.2	(19.9-26.8)	
Middle	69.8	(68.9-70.7)	56.7	(52.8-60.4)	
High	15.6	(15.1-16.3)	20.2	(17.4-23.2)	<0.001
**Employment status**					
Non-employed	18.9	(18.1-19.7)	32.6	(29.0-36.4)	
Part-time	30.3	(29.5-31.2)	19.7	(17.0-22.7)	
Full-time	50.8	(49.8-51.7)	47.7	(43.8-51.6)	<0.001
**Experienced unemployment**[c]					
No	90.0	(89.4-90.6)	90.7	(87.7-93.0)	
Yes	10.0	(9.4-10.6)	9.3	(7.0-12.3)	0.741
**Migration background**					
No	79.7	(78.8-80.5)	74.8	(71.0-78.4)	
Yes	20.3	(19.5-21.2)	25.2	(21.6-29.0)	<0.001
**Living with partner**					
No	8.8	(8.3-9.2)	38.1	(34.4-42.0)	
Yes	91.2	(90.8-91.7)	61.9	(58.0-65.6)	0.005
**Persons in household**					
1	0.0		0.0		
2	3.9	(3.7-4.2)	22.5	(19.4-25.9)	
3	33.4	(32.6-34.3)	26.0	(22.6-29.8)	
4	42.6	(41.7-43.6)	29.4	(25.8-33.3)	
5+	20.0	(19.2-20.9)	22.1	(19.0-25.6)	<0.001

[a] according to the CASMIN-classification; [b] net equivalent income compared to the median net equivalent income (low: under 60 %, middle: 60-150 %, high: > 150 %); [c] 12 months or more in the last 5 years

Supplementary material 2: Prevalence of risky alcohol consumption and heavy episodic drinking among non-parents according to selected covariates, n=45,203

	Risky alcohol consumption[a]				Heavy episodic drinking[b]			
	Women		Men		Women		Men	
	%	95% CI	%	95% CI	%	95% CI	%	95% CI
**Total**	23.0	(22.3-23.6)	34.1	(33.3-34.9)	9.9	(9.4-10.3)	24.1	(23.4-24.8)
**Age group**								
18-29	36.3	(34.7-38.0)	45.7	(44.0-47.4)	21.9	(20.6-23.3)	40.9	(39.3-42.6)
30-39	25.5	(23.0-28.1)	34.5	(31.9-37.1)	12.7	(10.9-14.8)	29.5	(27.1-32.0)
40-49	22.5	(20.7-24.5)	32.2	(30.0-34.5)	10.0	(8.7-11.5)	23.0	(21.0-25.0)
50+	19.5	(18.7-20.3)	30.0	(29.0-31.1)	6.6	(6.2-7.2)	17.1	(16.3-18.0)
p-value	<0.001		<0.001		<0.001		<0.001	
**Education[c]**								
Low	17.4	(16.3-18.5)	28.9	(27.5-30.4)	7.1	(6.4-7.9)	18.9	(17.7-20.2)
Middle	26.2	(25.3-27.1)	38.2	(37.1-39.4)	12.0	(11.4-12.7)	29.2	(28.1-30.3)
High	30.2	(28.8-31.6)	34.7	(33.4-36.0)	11.1	(10.1-12.1)	22.3	(21.2-23.5)
p-value	<0.001		<0.001		<0.001		<0.001	
**Income[d]**								
Low	20.3	(18.7-22.0)	32.4	(30.1-34.9)	9.5	(8.4-10.7)	24.8	(22.7-27.1)
Middle	21.9	(21.1-22.7)	33.1	(32.0-34.1)	9.5	(9.0-10.1)	22.8	(21.9-23.8)
High	29.8	(28.3-31.3)	37.6	(36.1-39.0)	11.7	(10.7-12.8)	26.8	(25.5-28.1)
p-value	<0.001		<0.001		0.001		<0.001	
**Employment status**								
Non-employed	19.9	(19.0-20.8)	30.3	(29.1-31.6)	7.5	(7.0-8.2)	19.3	(18.3-20.4)
Part-time	25.7	(24.3-27.1)	38.0	(35.5-40.6)	12.0	(11.0-13.1)	30.4	(28.0-32.8)
Full-time	27.7	(26.5-28.9)	36.7	(35.6-37.9)	13.4	(12.5-14.4)	27.2	(26.1-28.2)
p-value	<0.001		<0.001		<0.001		<0.001	
**Experienced unemployment[e]**								
No	25.9	(25.1-26.6)	36.3	(35.4-37.2)	12.2	(11.6-12.7)	27.1	(26.3-28.0)
Yes	20.9	(18.7-23.4)	32.4	(29.5-35.5)	7.9	(6.5-9.6)	23.5	(20.8-26.3)
p-value	<0.001		0.017		<0.001		0.017	
**Migration background**								
No	23.5	(22.8-24.2)	35.0	(34.1-35.9)	9.8	(9.3-10.3)	24.3	(23.5-25.1)
Yes	21.8	(20.0-23.8)	31.4	(29.0-33.8)	12.0	(10.5-13.7)	25.0	(22.8-27.3)
p-value	0.114		0.006		0.004		0.566	
**Living with partner**								
No	23.3	(22.4-24.2)	38.3	(37.1-39.6)	11.3	(10.7-12.0)	31.3	(30.1-32.4)
Yes	22.6	(21.7-23.6)	31.4	(30.4-32.5)	8.7	(8.1-9.4)	19.6	(18.7-20.5)
p-value	0.323		<0.001		<0.001		<0.001	

[a] AUDIT-C score (women: ≥ 4; men: ≥ 5); [b] ≥ 6 alcoholic drinks on a single occasion at least once a month; [c] according to the CASMIN-classification; [d] net equivalent income compared to the median net equivalent income (low: under 60 %, middle: 60-150 %, high: > 150 %); [e] 12 months or more in the last 5 years
